# Supplementary material for: A UMLS-based spell checker for natural language processing in vaccine safety
Source: BMC Med Inform Decis Mak. 2007 Feb 12;7:3. doi: 10.1186/1472-6947-7-3 (PMC1805499; doi:10.1186/1472-6947-7-3)
Supplement: Additional file 13 — RAP application directory. Sets up directory for RAP (RDF application for PHP) source code [file 1472-6947-7-3-S13.gz › rap/doc/phpdoc/default/FOAF.html]

xml version="1.0" encoding="iso-8859-1"?


Docs For Class FOAF


## Class FOAF

Description

Description |
Methods (details)

Located in /vocabulary/FOAF\_C.php (line 23)

Method Summary

Description |
Methods (details)

void
ACCOUNT\_NAME
()

void
ACCOUNT\_SERVICE\_HOMEPAGE
()

void
AGENT
()

void
AIM\_CHAT\_ID
()

void
BASED\_NEAR
()

void
CURRENT\_PROJECT
()

void
DEPICTION
()

void
DEPICTS
()

void
DNA\_CHECKSUM
()

void
DOCUMENT
()

void
FAMILY\_NAME
()

void
FIRST\_NAME
()

void
FUNDED\_BY
()

void
GEEKCODE
()

void
GENDER
()

void
GIVENNAME
()

void
GROUP
()

void
HOLDS\_ACCOUNT
()

void
HOMEPAGE
()

void
ICQ\_CHAT\_ID
()

void
IMAGE
()

void
IMG
()

void
INTEREST
()

void
JABBER\_ID
()

void
KNOWS
()

void
LOGO
()

void
MADE
()

void
MAKER
()

void
MBOX
()

void
MBOX\_SHA1SUM
()

void
MEMBER
()

void
MEMBERSHIP\_CLASS
()

void
MSN\_CHAT\_ID
()

void
MYERS\_BRIGGS
()

void
NAME
()

void
NICK
()

void
ONLINE\_ACCOUNT
()

void
ONLINE\_CHAT\_ACCOUNT
()

void
ONLINE\_ECOMMERCE\_ACCOUNT
()

void
ONLINE\_GAMING\_ACCOUNT
()

void
ORGANIZATION
()

void
PAGE
()

void
PAST\_PROJECT
()

void
PERSON
()

void
PERSONAL\_PROFILE\_DOCUMENT
()

void
PHONE
()

void
PLAN
()

void
PRIMARY\_TOPIC
()

void
PROJECT
()

void
PUBLICATIONS
()

void
SCHOOL\_HOMEPAGE
()

void
SHA1
()

void
SURNAME
()

void
THEME
()

void
THUMBNAIL
()

void
TIPJAR
()

void
TITLE
()

void
TOPIC
()

void
TOPIC\_INTEREST
()

void
WEBLOG
()

void
WORKPLACE\_HOMEPAGE
()

void
WORK\_INFO\_HOMEPAGE
()

void
YAHOO\_CHAT\_ID
()

Methods

Description |
Methods (details)

ACCOUNT\_NAME (line 98)

void

ACCOUNT\_NAME
()

ACCOUNT\_SERVICE\_HOMEPAGE (line 104)

void

ACCOUNT\_SERVICE\_HOMEPAGE
()

AGENT (line 26)

void

AGENT
()

AIM\_CHAT\_ID (line 110)

void

AIM\_CHAT\_ID
()

BASED\_NEAR (line 116)

void

BASED\_NEAR
()

CURRENT\_PROJECT (line 122)

void

CURRENT\_PROJECT
()

DEPICTION (line 128)

void

DEPICTION
()

DEPICTS (line 134)

void

DEPICTS
()

DNA\_CHECKSUM (line 140)

void

DNA\_CHECKSUM
()

DOCUMENT (line 32)

void

DOCUMENT
()

FAMILY\_NAME (line 146)

void

FAMILY\_NAME
()

FIRST\_NAME (line 152)

void

FIRST\_NAME
()

FUNDED\_BY (line 158)

void

FUNDED\_BY
()

GEEKCODE (line 164)

void

GEEKCODE
()

GENDER (line 170)

void

GENDER
()

GIVENNAME (line 176)

void

GIVENNAME
()

GROUP (line 38)

void

GROUP
()

HOLDS\_ACCOUNT (line 182)

void

HOLDS\_ACCOUNT
()

HOMEPAGE (line 188)

void

HOMEPAGE
()

ICQ\_CHAT\_ID (line 194)

void

ICQ\_CHAT\_ID
()

IMAGE (line 44)

void

IMAGE
()

IMG (line 200)

void

IMG
()

INTEREST (line 206)

void

INTEREST
()

JABBER\_ID (line 212)

void

JABBER\_ID
()

KNOWS (line 218)

void

KNOWS
()

LOGO (line 224)

void

LOGO
()

MADE (line 230)

void

MADE
()

MAKER (line 236)

void

MAKER
()

MBOX (line 242)

void

MBOX
()

MBOX\_SHA1SUM (line 248)

void

MBOX\_SHA1SUM
()

MEMBER (line 254)

void

MEMBER
()

MEMBERSHIP\_CLASS (line 260)

void

MEMBERSHIP\_CLASS
()

MSN\_CHAT\_ID (line 266)

void

MSN\_CHAT\_ID
()

MYERS\_BRIGGS (line 272)

void

MYERS\_BRIGGS
()

NAME (line 278)

void

NAME
()

NICK (line 284)

void

NICK
()

ONLINE\_ACCOUNT (line 50)

void

ONLINE\_ACCOUNT
()

ONLINE\_CHAT\_ACCOUNT (line 56)

void

ONLINE\_CHAT\_ACCOUNT
()

ONLINE\_ECOMMERCE\_ACCOUNT (line 62)

void

ONLINE\_ECOMMERCE\_ACCOUNT
()

ONLINE\_GAMING\_ACCOUNT (line 68)

void

ONLINE\_GAMING\_ACCOUNT
()

ORGANIZATION (line 74)

void

ORGANIZATION
()

PAGE (line 290)

void

PAGE
()

PAST\_PROJECT (line 296)

void

PAST\_PROJECT
()

PERSON (line 80)

void

PERSON
()

PERSONAL\_PROFILE\_DOCUMENT (line 86)

void

PERSONAL\_PROFILE\_DOCUMENT
()

PHONE (line 302)

void

PHONE
()

PLAN (line 308)

void

PLAN
()

PRIMARY\_TOPIC (line 314)

void

PRIMARY\_TOPIC
()

PROJECT (line 92)

void

PROJECT
()

PUBLICATIONS (line 320)

void

PUBLICATIONS
()

SCHOOL\_HOMEPAGE (line 326)

void

SCHOOL\_HOMEPAGE
()

SHA1 (line 332)

void

SHA1
()

SURNAME (line 338)

void

SURNAME
()

THEME (line 344)

void

THEME
()

THUMBNAIL (line 350)

void

THUMBNAIL
()

TIPJAR (line 356)

void

TIPJAR
()

TITLE (line 362)

void

TITLE
()

TOPIC (line 368)

void

TOPIC
()

TOPIC\_INTEREST (line 374)

void

TOPIC\_INTEREST
()

WEBLOG (line 380)

void

WEBLOG
()

WORKPLACE\_HOMEPAGE (line 392)

void

WORKPLACE\_HOMEPAGE
()

WORK\_INFO\_HOMEPAGE (line 386)

void

WORK\_INFO\_HOMEPAGE
()

YAHOO\_CHAT\_ID (line 398)

void

YAHOO\_CHAT\_ID
()

Documentation generated on Fri, 17 Dec 2004 16:14:53 +0100 by phpDocumentor 1.3.0RC3
